# Cynicism among medical students: An in-depth analysis of mental health dynamics and protective factors in medical education using structural equation modeling

**DOI:** 10.1371/journal.pone.0321274

**Published:** 2025-04-24

**Authors:** András Spányik, Dávid Simon, Adrien Rigó, Boróka Gács, Nóra Faubl, Zsuzsanna Füzesi, Mark D. Griffiths, Zsolt Demetrovics

**Affiliations:** 1 Doctoral School of Psychology, ELTE Eötvös Loránd University, Budapest, Hungary; 2 Institute of Psychology at ELTE Eötvös Loránd University, Budapest, Hungary; 3 Faculty of Social Science, ELTE Eötvös Loránd University, Budapest, Hungary; 4 Department of Behavioural Sciences, Medical School, University of Pécs,; 5 International Gaming Research Unit, Psychology Department, Nottingham Trent University, Nottingham, United Kingdom; 6 Flinders University Institute for Mental Health and Wellbeing, College of Education, Psychology and Social Work, Flinders University, Adelaide, South Australia, Australia; 7 Centre of Excellence in Responsible Gaming at the University of Gibraltar, Gibraltar; Guangxi Normal University, CHINA

## Abstract

**Background:**

Medical students frequently grapple with challenges during their studies, including emotional impacts, career socialization, psychiatric comorbidities, and burnout syndrome. Burnout syndrome profoundly influences mental and physical health, impacting patient care. Within this complex landscape, elevated stress levels specifically manifest in increased cynicism, reduced idealism, and other mental health issues. The simultaneous decline of empathy during medical education adds a layer of complexity. Understanding these dynamics and the potential protective factors is crucial for addressing students’ well-being and optimizing curriculum development.

**Methods:**

The present study comprised third-year and fifth-year Hungarian medical students from the University of Pécs in a partially cross-sectional, partially longitudinal investigation conducted at two time points (2018/19: 124 third-years; 127 fifth-years; 2020/21: 82 third-years; 37 fifth-years). All medical students were sampled in the given year of the selected semester (third-year students at the first timepoint were asked once again as fifth-year students at the second timepoint). In addition to descriptive statistics, structural equation modelling was used to assess the impact of time, role model, perceived stress, empathy, and burnout on medical students.

**Results:**

The analysis indicated that there was a significant increase in cynicism during the institutional socialization of medical students. While there was only partial support for the reduction in the perception of patient-centered role models during institutional socialization, the findings indicated that the perception of patient-centered role models and empathy acted as protective factors mitigating cynicism. Unexpectedly, the analysis found an increase in stress and cynicism over time, possibly influenced by factors such as the COVID-19 pandemic.

**Conclusion:**

The study’s findings indicate a rise in cynicism among medical students over time that could threaten future doctor-patient relationships. The findings emphasize the protective role of empathy and patient-centered role models, emphasizing the need for humanistic integration in medical education.

## Introduction

The adverse effects medical students are faced with during their studies have been widely studied [1]. Among others, these studies with medical students have addressed issues such as the emotional effects of the training procedure and career socialization, which can have a significant impact on their mental and physical health [1,2]. Other relevant factors that can affect medical students’ education include psychiatric comorbidities, such as sleep disorders, alcohol and drug addictions, and the increased prevalence of anxiety and depression [3,4].

Another negative consequence associated with working in the medical profession is burnout syndrome, a condition that can emerge during education before commencing professional practice [5–7]. Burnout is characterized by a triad of emotional exhaustion, depersonalization, and a decreased sense of accomplishment and has been associated with higher suicidal ideation among medical students [8]. The consequences of burnout syndrome are profound and extend beyond its impact on the mental health of medical students. It also affects their physical health and capacity to deliver optimal patient care during medical school and future medical practice [9].

According to Heinen et al. [10], the higher prevalence of burnout levels among medical students compared to the general population may be due to the significantly increased level of perceived stress [5,7,11]. In addition to a large amount of coursework and explicit examination requirements, less adequate coping strategies also contribute to stress [12,13]. As a result of increased stress exposure during medical training, an increase in the prevalence of cynicism, decreased idealism, and other mental health problems among students have been reported [12,14].

The Maslach Burnout Inventory Student Survey (MBI-SS) is the gold standard for assessing student burnout [15]. It comprises three subscales: emotional exhaustion, cynicism, and reduced personal accomplishment. It is important to note that within the constructs evaluated by the MBI, the cynicism subscale holds significant importance in the context of the present study because it is a strong predictor of burnout symptoms. The cynicism dimension was originally referred to as depersonalization and encompassed irritability and a negative, inappropriate attitude towards other people in the workplace [16]. A scoping review identified increasing cynicism during medical education as a significant issue, highlighting its negative impact on future doctor-patient relationships and physicians’ mental health. Additionally, it suggests that cynicism may also function as a defense mechanism against more severe reactions, such as depression [17]. This dimension captures a critical aspect of healthcare, which is clearly of significant importance to the delivery of quality care [15].

Moreover, cynicism plays a vital role in workplace social interactions and interpersonal relationships, making it an important factor in relational dynamics [15]. The importance of cynicism during medical studies is also supported by the observation that higher cynicism rates are associated with inappropriate doctor-patient relationships during medical practice and unsatisfied patients and have an adverse effect on patient compliance [18]. Research suggests that the elevated levels of cynicism reported among medical students can be explained by heightened stress, inappropriate coping strategies acquired during medical career socialization, and a decrease in empathy [5,7]. In medical education, cynicism is perhaps the most heavily influenced dimension in the context of professional attitudes and interpersonal dynamics and plays a crucial role in the development of the doctor-patient relationship. It is also of substantial importance in physicians’ mental health, highlighting its multidimensional impact [19]. Cynicism has been identified as a significant factor in high workplace turnover and job dissatisfaction, which are undoubtedly among the most critical challenges facing education and healthcare management [20]. Therefore, addressing and reducing cynicism is clearly an essential task [16].

According to a comprehensive literature review [21], the degree of empathy exhibited by medical students tends to diminish throughout their medical education, and this decline persists throughout their residency training. The complexity of factors influencing empathy during medical education adds an additional layer to the challenges faced by medical students [22,23].

A growing body of research has established the importance of physician empathy in improving patient satisfaction, adherence to therapy, clinical outcomes, and reducing malpractice liability [6,22]. Therefore, investigating changes in empathy during medical studies is crucial [6,22]. In academic discourse, empathy concepts are commonly delineated through two primary classifications: vicarious empathy and imaginative empathy [6]. Vicarious empathy refers to the phenomenon wherein an individual elicits an emotional response in response to the distress or emotional experiences of another individual [24]. Imaginative empathy encompasses a cognitive capacity through which individuals can comprehend the emotional states of others, immerse themselves in their circumstances, and prognosticate their emotions and intentions [25]. This decline in empathy and the increased level of cynicism can be partly traced back to the profound influence of the implicit and hidden curriculum. This emphasizes the pivotal role of these covert educational factors in shaping the empathic development of medical professionals [22].

Numerous empirical studies have investigated the impact of the implicit curriculum and the hidden curriculum on medical education [22,26]. The implicit curriculum refers to the knowledge and skills that students gain from observing their mentors during their time in college (for example, specific type of doctor-patient role models). The hidden curriculum refers to the organizational and institutional factors that shape the education of students in subtle ways [27].

As a theoretical background, social learning theory [28] emphasizes that individuals learn behaviors, attitudes, and norms by observing and emulating role models. Patient-oriented role models who demonstrate empathy, effective communication, and ethical patient care provide positive behaviors for students, and can counteract the development of cynical attitudes. Based on their in-depth qualitative research, Passi and Johnson [29] highlighted the importance of patient-centered role models in reinforcing professionalism and professional identity.

Understanding and addressing the consequences of adverse mental health effects during medical studies are crucial for developing comprehensive strategies to tackle students’ challenges effectively, ensuring their well-being throughout their education and future medical practice [8,30]. Moreover, it is imperative to investigate changes in empathy and explore the association between empathy and adverse mental health effects, adding depth to the understanding of the challenges faced by medical students [6,22]. The examination of the impact of the curriculum is of paramount importance in line with the aforementioned considerations because it is presumed to be instrumental in influencing empathy and, consequently, other mental health issues [22,27].

### Aims and hypotheses

**Fig 1:**
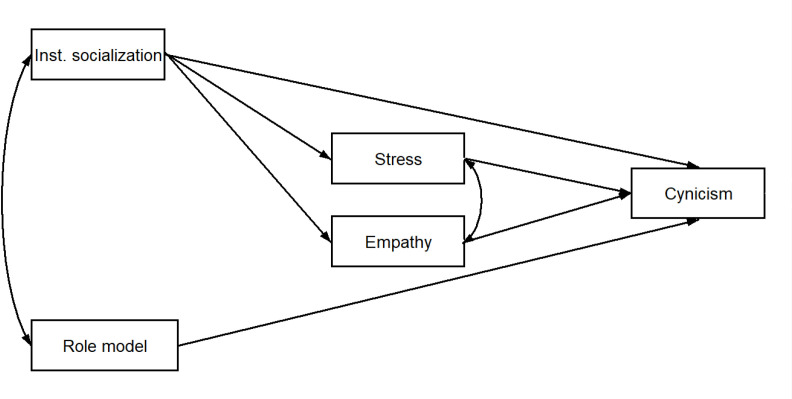
Hypothetical model of relationship between (length of) institutional socialization, role model, stress, empathy and burnout.

Given the findings in the aforementioned literature, the aim of the present study was to investigate the changes in empathy, stress, and cynicism of medical students during their training and to examine how these psychological factors are interrelated. The study also aimed to determine the impact of the role model as part of implicit curriculum on these aforementioned changes. Fig 1 provides a visual representation of the hypothetical model that illustrates the relationship among these factors. Beyond this model, hypotheses (H_s_) were developed related to the temporal evolution of different psychological factors. More specifically, it was hypothesized that:

- H_1_: Stress levels among medical students would rise over the course of their medical education (based on studies showing medical students have increased levels of stress during their medical training [e.g., 5, 7, 11]).
- H_2_: Empathy levels among medical students would decline gradually over the course of their medical education (based on the systematic review by Ferreira-Valente et al. [21]).
- H_3_: The level of cynicism among medical students would increase over the course of their medical education (based on multiple previous studies reviewed by Hershey et al. [17]).

Moreover, hypotheses concerning the relationships between these aforementioned factors were also developed. More specifically, it was hypothesized that:

- H_4_: Empathy and the role model would have a mitigating effect on cynicism among medical students (based on the [i] possible mitigating effect of empathy on cynicism [5,7]), and [ii] mitigating effect of role model on cynicism applying social learning theory [28], and empirical findings [29]).
- H_5_: Higher stress levels would be positively associated with increased cynicism among medical students (based on empirical studies by Dyrbye et al. [5] and Peng et al. [7]).

The study’s theoretical model also included the remaining part of the hidden and implicit curriculum (institutional socialization) in order to compare it to the measured effect of the role model. The stability of the results was also investigated by assuming that H_1_-H_4_ would be independent of the timepoint of data collection (H_6_). Inclusion of the timepoint of data collection also made it possible to eliminate the effect of external events. These hypotheses provide a framework for exploring the dynamics of psychological factors and their interrelationships within the context of medical education.

## Methods

### Participants and context

Third-year and fifth-year Hungarian medical students at the University of Pécs were invited to participate in a repeated cross-sectional study. At the Faculty of General Medicine at the University of Pécs, during the six-year program, the most important knowledge related to the doctor-patient relationship is taught within the framework of behavioral science subjects. These subjects are covered in the first five semesters of the curriculum. The curriculum includes medical anthropology, medical sociology, medical psychology, and medical communication. Additionally, students can participate in Balint groups as part of a student-organized program, and they also have access to free psychological consultations and peer support groups.

Data collection took place at two time points comprising a mixture of cross-sectional and longitudinal data. First, during the first semester of the 2018/2019 academic year (T1) and approximately two and a half years later, during the second semester of the 2020/2021 academic year (T2). Third-year and fifth-year students were selected as the target population, after the subjects related to behavioral science alongside regular practice in hospitals had been completed. Final year students were excluded due to technical reasons because there are no regular practices where the measurement would have been possible.

The final sample comprised 251 individuals in 2018/2019 (124 third-year students: 47.6% man and 52.4% women, mean age of 22.1 years; 127 fifth-year students: 34.6% men and 65.4% women, mean age of 24.0 years) and 119 individuals in 2020/2021 (among them 82 third-year students and 37 fifth-year students). In the second wave of data collection, those students in the fifth year comprised the same cohort that were third-year students from the previous wave of data collection.

### Procedure

American Psychological Association ethical standards and the principles outlined in the Helsinki Declaration were followed, and the study was approved by the Research Ethics Committee of the Faculty of Pedagogy and Psychology of Eötvös Loránd University (2018/168). All participants gave their written informed and voluntary consent for participation and could withdraw it at any time. The survey was pretested and adjusted in a pilot with medical students. In the first phase of the research, in 2018, the medical students completed the survey offline (i.e., paper-and-pencil) within the framework of their academic exercises belonging to the curriculum (the first data collection was conducted between September 1, 2018 and December 7, 2018). Due to the COVID-19 pandemic, an online survey was used in the second wave of data collection because students were not generally on-site (this data collection was conducted between November 13, 2020 and June 28, 2021). Students were asked to complete the online survey during or after their online course on their curriculum, which is a possible reason for the lower response rate compared to the first phase of data collection.

### Measures

Elements of the conceptual model were partially assessed using self-developed measures (i.e., positive role model and institutional socialization). Other elements were assessed by standardized and validated psychometric scales. More specifically, stress was assessed using the Perceived Stress Scale and the ‘Personal Distress’ subscale of Interpersonal Reactivity Index (IRI). Empathy was assessed using the ‘Empathic Concern’ subscale of the IRI. Cynicism was assessed using the ‘Cynicism subscale’ of Maslach Burnout Inventory (MBI).

#### Role model.

The effect of role model was assessed by the perception of patient-centered doctor-patient relationship role models with a self-developed item that was used as a proxy variable. This item was based on the mutual type of doctor-patient relationship according to Stewart and Roter’s [31] model. The item assessed the extent to which the participant perceived patient-centered doctor-patient relationship based on two specificities of the aforementioned type of relationship (partnership and information sharing) among their tutors, assuming that the two characteristics of the type of relationship coexist most of the time (*“The patients are considered partners by tutors and patients are informed about all the important details of their treatment”*). The item was scored on a seven-item scale from 1 (“*not typical at all”*) to 7 (“*completely typical”*).

#### Institutional socialization (complex effect of medical training).

The effect of institutional socialization was assessed in the models by the number of years the students had spent in the training (measured by which year of the course the student was in). This variable comprises all of the unmeasured effects related to institutional socialization.

#### Stress and distress.

Two instruments were used for assessing stress: Interpersonal Reactivity Index – Personal Distress subscale (IRI-PD) and the Perceived Stress Scale (PSS). Assessing stress was a key element of the study. Using two different instruments was likely to increase the reliability and validity of the findings. Moreover, the two scales assess different aspects of stress. The PSS assesses the general and intrapersonal element, whereas the IRI-PD assesses the interpersonal element of stress.

Perceived stress was assessed using the 14-item version of the PSS ([32]; Hungarian version: [33]) comprising seven positive and seven negative items. Each item (e.g., *“In the last month, how often have you felt that you were unable to control the important things in your life?”*) is scored on a five-point scale from 0 (*never*) to 4 (*very often*), with positive items reverse scored (scale range: 0–56; Cronbach alpha:.88).

Personal distress was assessed using the Personal Distress subscale of the IRI ([25]; Hungarian version [34]). The IRI has four subscales: Perspective Taking (IRI-PT) assessing the tendency to adopt the psychological viewpoint of others; Fantasy (IRI-FS), assessing the tendency to emphasize a fictitious character of a literary work; Empathic Concern (IRI-EC) assessing the feeling of warmth and concern for others; and Personal Distress (IRI-PD) assessing the anxiety regarding other’s negative experiences. The subscales each include seven items, of which various items are negative while the others are positive. Each item (e.g., *“Being in a tense emotional situation scares me”*) is scored on a five-point scale from 0 (*does not describes me at all*) to 4 (*describes me very well*) with positive items reverse scored (scale range: 0–28, Cronbach alphas:.73,.79,.70,.74 respectively).

#### Empathy.

Empathy was assessed using the Empathic Concern subscale of the IRI (IRI-EC) assessing the feeling of warmth and concern for others.

#### Cynicism.

Cynicism was assessed using the ‘Cynicism’ subscale of Maslach Burnout Inventory (MBI) for Students ([35,36]; Hungarian version [37]). The MBI has three subscales assessing different dimensions of burnout: Emotional Exhaustion subscale (MBI-EE; five items), Cynicism subscale (MBI-CY; four items), and Professional Efficacy subscale (MBI-PE; six items). Items (e.g., *“I doubt the significance of my studies”*) are scored on a seven-point scale from 0 (*never*) to 6 (*always*) (subscale ranges: 0–30, 0–24, 0–36, Cronbach alphas:.84,.85,.83 respectively).

#### Effect of time.

The year of data collection was used as a control variable.

### Statistical analysis

Descriptive statistics were calculated for all variables of the study (i.e., means and standard deviations). Student *t*-tests were used with Sidak correction examining the difference between means of each class of each time-point of research. All variables were considered to be nearly normally distributed if skewness and kurtosis were in the range of +/-2 [38]. All variables had skewness values between -.41 and.44, and kurtosis values between -.85 and.21. Cronbach’s α reliability estimation was conducted for psychometric scales. Correlation analysis was conducted by computing Pearson’s correlation coefficients with two-tailed significance tests. A *p*<.05 significance level was used for all statistical tests. The strength of correlation was assessed according to Cohen [39]. SPSS v.23 was used for the descriptive statistics, reliability analysis, and correlation analysis.

The hypotheses were tested using structural equation modelling. The estimation method was selected according to normality check of the participating variables. All paths that were not significant (*p*>0.05) were removed from the model. The goodness of fit of the model was tested by likelihood ratio tests (model versus baseline, model versus saturated), root mean square error approximation (RMSEA), comparative fit index (CFI), Tucker-Lewis index (TLI), and goodness of fit index (GFI). A model is considered to have a ‘good’ fit if RMSEA <0.05 and acceptable if it is <0.08. In the case of TLI, CFI, and GFI, >0.90 is considered acceptable [40]. Unstandardized and standardized coefficients as well as total effect (calculated from direct and indirect effect) and equation-level goodness of fit (R^2^) were calculated for the final model.

## Results

### Descriptive statistics and preliminary analyses

Table 1 provides descriptive statistics of the study variables and their differences according to length of institutional socialization and timepoint of data collection. Significantly lower patient-centered role model perception was found among fifth-year students compared to third-year students in 2018/2019. That was neither the case in 2020/2021 nor between 2018/2019 and 2020/2021 in case of the following cohort.

**Table 1:** Changes in descriptive statistics of the study variables over time and between year classes.

	2018/2019						2020/2021						Total				
	Third-year			Fifth-year			Third-year			Fifth-year							
Variables	n	M	SD	N	M	SD	n	M	SD	n	M	SD	n	M	SD	Skewness	Kurtosis
1. Patient-centered Role Model perception	121	4.93_ab_	1.32	126	4.44_ba_	1.49	79	4.95_ab_	1.22	37	4.73_ab_	1.02	363	4.74	1.35	-.14	-.33
2. PSS	122	27.32_ab_	8.65	126	26.33_ab_	8.00	73	31.11_ba_	8.99	34	29.97_ab_	9.96	355	28.00	8.80	.19	-.12
3. IRI- Perspective taking	124	18.06_ab_	4.62	125	17.12_ab_	4.33	79	19.25_ba_	4.88	36	18.33_ab_	4.86	124	18.02	4.65	-.20	-.45
4. IRI- Fantasy	124	17.82_ab_	5.37	126	16.89_ab_	5.79	79	19.54_ba_	5.41	36	17.69_ab_	5.92	124	17.86	5.65	-.31	-.32
5. IRI- Personal distress	124	11.97_ab_	4.78	127	11.04_ab_	4.92	79	11.49_ab_	5.59	35	11.14_ab_	6.46	365	11.46	5.18	-22	-.33
6. IRI- Empathic concern	123	17.43_ab_	5.3	125	17.86_ab_	4.10	79	18.85_ab_	4.54	35	17.77_ab_	5.31	362	17.92	4.75	-.18	-.24
7. MBI- Emotional exhaustion	118	14.19 _ab_	7.04	114	15.12_ab_	7.08	80	17.35_ba_	7.68	37	17.86_bc_	7.39	118	15.61	7.34	.07	-.70
8. MBI- Cynicism	118	7.16_ab_	6.12	115	9.52_ba_	6.72	80	9.33_ab_	6.24	37	12.57_ba_	6.74	350	9.00	6.59	.44	-.85
9. MBI- Professional efficacy	117	24.03_ab_	6.62	115	25.07_ab_	6.27	80	23.19_ab_	6.75	37	23.11_ab_	6.70	117	24.08	6.57	-.41	.21

N otes: Means for different timepoint and different class in the same row not sharing the same subscript were significantly different at *p*<.05 in the two-sided test of equality for column means. Cells with no subscript were not included in the test. Tests assumed equal variances. PSS = Perceived Stress Scale; IRI = Interpersonal Reactivity Index; MBI = Maslach Burnout Inventory

There was no significant difference in perceived stress between third-year and fifth-year either in one year or in the following cohort. This finding did not support H_1_. However, perceived stress was found to be significantly higher in the third-year in 2020/2021 compared to both the third-years and fifth-years in 2018/2019. Different aspects of empathy followed a similar pattern both over time and in relation to institutional socialization. Scores were not significantly different either between classes in the same time points or between time points in the same cohort. The only significant difference occurred in scores of IRI-PT and IRI-FS between the fifth-year in 2018/19 and third-year in 2020/2021. These findings did not support H_2_.

A larger heterogeneity was found in the aspects of burnout. In the case of MBI-PE, there was no significant difference either in time or in relation with length of institutional socialization. On the other hand, MBI-EE was significantly higher in the third-year in 2020/2021 than in the third-year in 2018/2019, while no other significant differences were found in this dimension. In the case of cynicism, a significantly higher level was found in the fifth-year compared to the third-year in 2018/2019, and an increase in the following cohort between 2018/2019 and 2020/21. This result supported H_3_. Table 2 provides Pearson correlations between all study variables. Patient-centered role model had a significant (i) weak to moderate positive correlation with IRI-PT and MBI-PE, and (ii) significant but weak negative correlation with MBI-EE and MBI-CY. The latter finding supports H_5_.

**Table 2:** Pearson correlations for study variables.

Variables	n	1	2	3	4	5	6	7	8	9
1. Patient-centered Role Model perception	363	–								
2. PSS	355	-,10^**^	–							
3. IRI- Perspective taking	364	.13^**^	.00^**^	–						
4. IRI- Fantasy	365	.00^**^	.21^**^	.27^**^	–					
5. IRI- Personal distress	365	.00^**^	.47^**^	-.06^**^	.09^**^	–				
6. IRI- Empathic concern	362	.05^**^	.14^**^	.39^**^	.41^**^	.17^**^	–			
7. MBI- Emotional exhaustion	349	-.11^**^	.59^**^	.00^**^	.07^**^	.32^**^	.07^**^	–		
8. MBI- Cynicism	350	-.18^**^	.36^**^	-.06^**^	-.03^**^	.23^**^	-.15^**^	.64^**^	–	
9. MBI- Professional efficacy	349	.20^**^	-.42^**^	.13^**^	.04^**^	-.32^**^	.19^**^	-.39^**^	-.49^**^	–

Notes:

* *p*<.05,

** *p*<.01. PSS = Perceived Stress Scale; IRI = Interpersonal Reactivity Index; MBI = Maslach Burnout Inventory

Perceived stress had a significant moderate to strong correlation with the subscales of empathy and the subscales of burnout, except for IRI-PT which was not correlated with PSS at all. PSS had a negative correlation with MBI-PE only. These findings supported H_5_. There were significant, mostly moderate, positive correlations between the dimensions of empathy except between IRI-PT, IRI-FS, and IRI-PD. The correlations between dimensions of burnout were strong or nearly strong and positive between MBI-EE and MBI-CY, whereas they were negative between MBI-EE, MBI-CY, and MBI-PE. Among the dimensions of empathy, only IRI-PD and IRI-EC were significantly correlated to dimensions of burnout with weak to moderate strength. The correlations between IRI-EC and MBI-CY as well as the correlation between IRI-PD and MBI-EE were negative, while other correlations between subscales of empathy and burnout were positive where they were significant.

The decision was made to exclude MBI-PE and MBI-EE from the model based on the lack of coherent significant differences over time or between classes. Similarly, IRI-FS and IRI-EC were excluded from the model due to their lack of significant correlation with burnout and role models, as well as the absence of significant differences over time and between classes.

### Structural equation model

Maximum likelihood model with missing values was used for fitting the model because all criteria were met. The initial model did not fit the data: ***χ***^**2**^=14.33 (df=4) *p*=.006; CFI=.95; TLI=.77; GFI=.94, RMSEA=.084 (90% CI:.040,.132), AIC=11865.8. Modification of the original model based on modification indices and removing the non-significant paths led to the final model. The final model fitted the data: ***χ***^**2**^=15.12 (df=8) *p*=.057; CFI=.97; TLI=.92; GFI=.94, RMSEA=.049 (90% CI:.000,.087), AIC=11848.6. The overall coefficient of determination of the model was.21.

**Fig 2:**
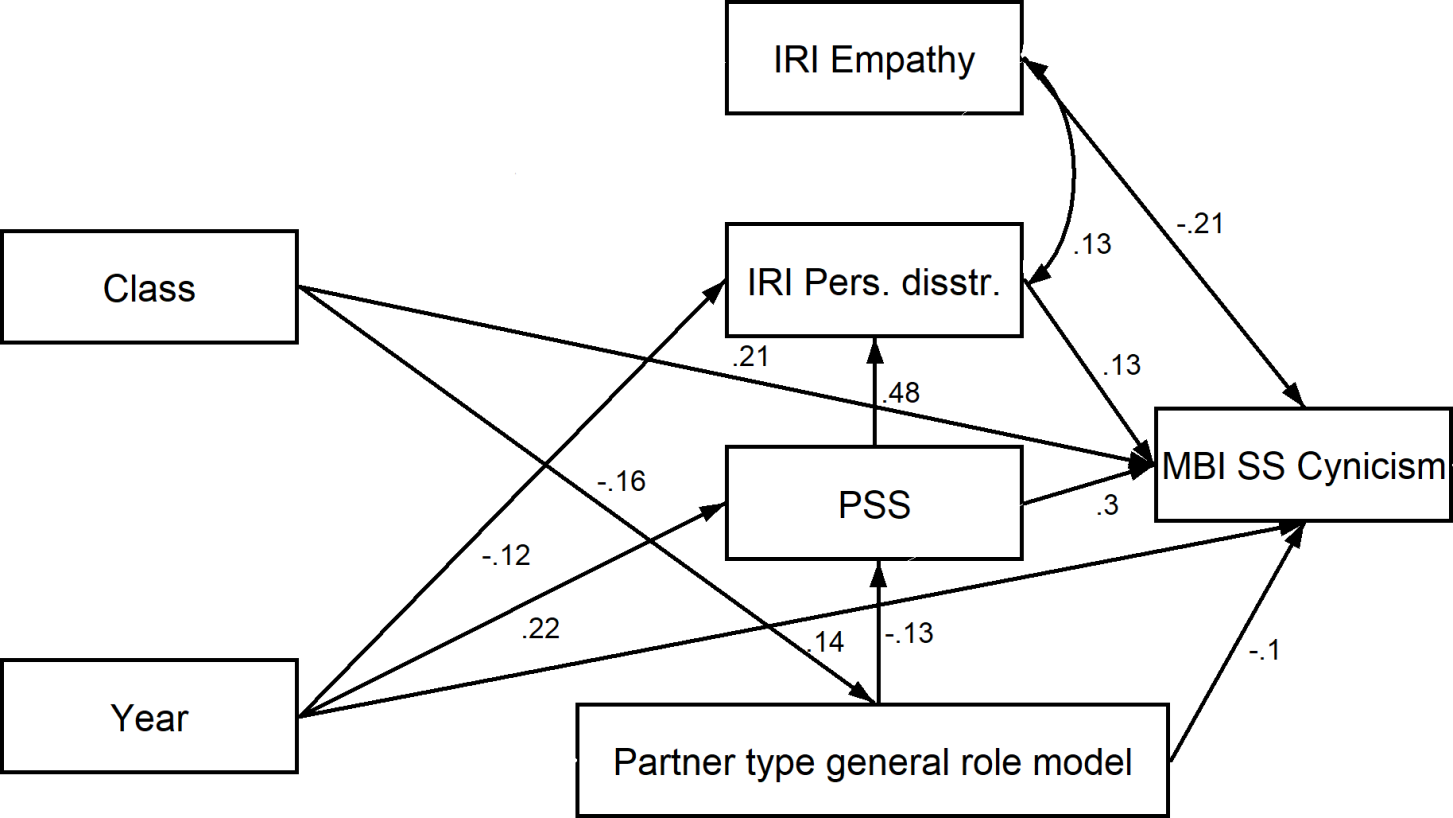
Relationship between patient-centered role model perception, time spent in medical education, timepoint of investigation, stress, empathic concern, and cynicism (final SEM model).

In the final SEM model (Fig 2), the length of medical education (class) had a positive but weak direct effect on cynicism and a negative but weak direct effect on the perception of partner-type role models. Time-point of data collection had a weak to intermediate effect on perceived stress, a weak negative effect on personal distress, and a weak but positive effect on cynicism. Partner-type role model had weak negative effect both on perceived stress and cynicism. Perceived stress had a moderate to strong positive effect on cynicism and personal distress. Personal distress had a weak positive effect on cynicism. Empathic concern had a weak to moderate negative effect on cynicism and a weak positive effect on personal distress.

Assessing the total effects of each of the investigated factors (Table 3), in the case of cynicism a moderate to strong positive effect of perceived stress was found while personal distress had an independent weak positive effect. These findings supported H_5_. On the other hand, perceived partner type of doctor-patient role model and empathic concern had a weak to moderate decreasing total effect on cynicism. This result supported H_4_. The length of time spent in medical education and the time-point of data collection had a weak to moderate positive total effect on cynicism. The effect of time spent in medical education supported H_3_. However, a significant independent effect of time-point did not support H_6_.

**Table 3:** Total effects of time spent in medical education, timepoint of investigation, patient-centered role model perception on stress, empathic concern and cynicism.

		Std. Coeff.	z	*p*>|z|
Cynicism	Perceived stress	.36	6.66	.000
	Personal distress	.13	2.49	.013
	Empathic concern	-.21	-4.42	.000
	Patient centered role model	-.16	-2.99	.003
	Class	.20	3.80	.000
	Year	.17	3.31	.001
Personal distress	Perceived stress	.49	10.21	.000
	Patient centered role model	-.07	-2.76	.006
	Class	-.08	-1.41	.158
	Year	-.02	-0.44	.663
Perceived stress	Patient centered role model	-.14	-2.76	.006
	Class	.07	-1.25	.211
	Year	.20	3.68	.000
Patient centered role model	Class	-.16	-2.97	.003
	Year	.04	0.70	.487

Empathy was found to be independent of both the length of time spent in medical education and the time point of data collection. Therefore, this did not support H_3_. In the case of personal distress, a strong positive total effect of perceived stress was found. Neither the length of time spent in medical education, nor time-point of data collection had a significant total effect at all on personal distress. Regarding perceived stress, a weak negative total effect of perceived patient-centered role model was found, while time-point of data collection was found to be not significant. The total effect of time spent in medical education had a weak positive total effect. This latter finding supported H_2_. Patient-centered role model had a statistically significant but very weak negative total effect on personal distress mediated by perceived stress. Moreover, time spent in medical education had a weak but significant negative effect on the perception of patient-centered role model.

## Discussion

The present part-cross-sectional, part-longitudinal study examined the interplay of empathy, stress, and cynicisms among medical students through their training, and investigated the influence of institutional socialization, and role models on these psychological factors. The findings demonstrated a significant increase in cynicism during the institutional socialization of medical students (supporting H_3_). Multiple studies have observed a substantial rise in cynicism levels among medical students, possibly attributed to their placement at the lower echelons of the hierarchical system with limited control and increased vulnerability during practical courses [41]. Moreover, although the analysis showed partial reduction in the perception of patient-centered role models during institutional socialization (H_1_), the findings indicated that the perception of patient-centered role models and empathy served as protective factors mitigating cynicism (supporting H_4_).

While it was assumed that all effects related to the training would be stable over time (and therefore no significant effect of the time of data collection was hypothesized), the analysis indicated that the time of data collection had a significant effect in the case of stress and cynicism which both independently increased from the first wave to the second, independently of the time spent in education. The reason for this unexpected result is not clear, but a possible effect of the COVID-19 pandemic cannot be ruled out. This is further discussed below in relation to limitations of the research.

According to previous research, increased cynicism during the curricula may also be explained by the lack of positive (patient-centered) role models during medical training and the hierarchical nature of the medical education system [19,41,42]. Additionally, medical students encounter coping mechanisms typical of the clinical stage, but often, the dismissive attitude of residents and healthcare professionals negatively impacts medical students’ attitudes [41].

Empathy may exert a negative (protective) influence on cynicism, potentially tied to the attitudes developed by students in the course of their medical education – a factor that, as evidenced by prior research, has also been suggested to correlate with the selection of medical specialization [43]. According to existing studies, ‘people-oriented’ students tend to exhibit higher levels of empathy and lower levels of cynicism, while ‘technology-oriented’ students tend to show lower levels of empathy and higher levels of cynicism [43].

However, contrary to H_1_ and H_2_, the analysis did not find evidence supporting an increase in stress or a decrease in empathy among medical students. A scoping review indicated that most longitudinal studies have yielded mixed results or reported declines in empathy among medical students [21]. The ultimate conclusion drawn from the present study suggests that, presently, the existing literature needs more definitive conclusions regarding changes in student empathy throughout medical school [21]. Similar to empathy, there is a need for longitudinal studies examining stress with consistent findings [44]. Nevertheless, most studies indicate that students’ stress levels are significantly higher than the general population [10].

Conversely, the present study’s findings indicated that perceived stress and personal distress were associated with an increase in the level of cynicism. These results are in line with several studies that provide support for H_6_, indicating that substantial stressors encountered during medical education play a role in the heightened cynicism levels observed throughout medical training [12,14].

### Limitations

The present study had a number of limitations. The effect of role model was assessed using a self-developed item. It is also important to acknowledge that the second phase of data collection took place during the COVID-19 pandemic. The pandemic caused significant emotional distress to medical students, adding an element of uncertainty because many aspects of their education were limited or conducted online [45]. Moreover, upper-level students were called upon to contribute to public health efforts (for example, screening potentially infected people) [45]. These circumstances could have negatively impacted students’ stress levels and led to burnout [46]. Additionally, the effects of career socialization may have been altered due to limited interactions with instructors and atypical patient encounters [47].

The pandemic’s uncertainty, combined with concerns about the virus, could also have served as additional stressors [47]. Based on these considerations, it is possible that the variability in the effects may be due (in part) to the impact of the COVID-19 pandemic, given that the proposed model indicated that the levels of perceived stress, personal distress, and cynicism were contingent upon the timing of data collection, irrespective of the student’s academic year of study. The pandemic also necessitated adjustments to the data collection methods, leading to a reduced response rate during the second data collection phase and preventing matching data at a personal level. Consequently, the analytical capabilities were constrained by these changes. Other limitations of the study include the collection of data from students of a single institution, limiting generalizability and the exploration of institutional effects. Additionally, the small convenience sample also hinders generalizability. Moreover, the potential biases of self-reported data may have impacted data quality (e.g., social desirability bias, memory recall bias). Future research needs to replicate the study with a larger and more representative sample during a period of lower national disruption.

## Conclusion

The present study explored the impact of medical training on empathy and burnout among medical students. The findings showed a significant increase in cynicism over time, potentially endangering future doctor-patient relationships and patient outcomes. Notably, the study highlights the protective role of empathy and exposure to patient-centered role models in mitigating cynicism, which tends to arise from an excessive focus on technical skills and biomedical aspects at the expense of humanistic elements in medical care. The observed rise in cynicism appears to be associated with students’ disillusionment in their medical roles. These results emphasize the necessity of integrating humanistic aspects into medical education and providing emotional support mechanisms for students to prevent burnout and enhance doctor-patient relationships. As international examples show, with a well-designed intervention, students’ empathy can be improved, and stress can be reduced if the environment is appropriate [48]. The study outcomes could serve as valuable insights for curriculum development, student support initiatives, and future research endeavors.
